# A Calibration Device to Compare Body Plethysmographs Among Pediatric Lung Function Laboratories

**DOI:** 10.3389/fphys.2018.01408

**Published:** 2018-10-09

**Authors:** Bruno Demoulin, Iulia Ioan, Claude Duvivier, Claude Bonabel, Cyril Schweitzer, François Marchal, Silvia Demoulin-Alexikova

**Affiliations:** ^1^EA 3450 DevAH, Department of Physiology, Faculty of Medicine, University of Lorraine, Nancy, France; ^2^Department of Pediatric Respiratory Function Testing, Children’s Hospital, Vandoeuvre, France; ^3^Departement of Pediatrics, Children’s Hospital, Vandoeuvre, France

**Keywords:** plethysmography, pediatric respiratory disease, thoracic gas volume, collaborative studies, airway resistance

## Abstract

Multi-center studies in specific airway resistance have shown significant inter laboratory variability. Comparison of plethysmographic equipment using a lung model easily transportable from one site to another should be of help to international normative studies. A resistor made of parallel capillary tubes – insuring adequate linearity within 1 L/sec – was connected to a glass bottle. Thermal time constants were measured while the bottle was empty and while stuffed with steel wool. In the latter, isothermal condition was estimated to occur only at very low frequency (around 0.01 Hz) and gas compression was polytropic up to 0.6 Hz. With the empty analog, adiabatic gas compression was estimated to occur at frequencies ≥0.2 Hz, and more accurate volume estimation was obtained. The empty analog volume and specific resistance measured in a body plethysmograph on different days indicated within 5% accuracy as well as intersession repeatability. It is concluded that a physical analog built out of simple material provides accurate measurements of specific resistance. The apparatus should be of help to compare plethysmographic equipments from different laboratories.

## Introduction

International collaborative studies are important to better understand, assess and manage pediatric respiratory diseases such as asthma, chronic lung disease of prematurity or cystic fibrosis. Lung function provides invaluable objective assessment of the outcome. It is of primary importance that standardized procedures are used and equipment measures similarly among and across centers. Procedures may be formulated and standardized at international workshop conferences, and it is a matter of discussion and communication to delineate recommendations and guidelines based on experience sharing and literature review (Stocks and Quanjer, 1995; Miller et al., 2005; Beydon et al., 2007). Equipment type and/or specifications often differ among laboratories and between-center agreement has to be tested for optimal interpretation.

The plethysmographic measurement of specific airway resistance (sRaw) has gained popularity in preschool children during the last few years as it is achievable with minimal subject cooperation (Bisgaard and Klug, 1995; Klug and Bisgaard, 1997). Collaborative studies have revealed significant center dependence of sRaw (Kirkby et al., 2010; Paton et al., 2012), equipment related variability, and within a commercial brand, dependence on the software version itself (Kirkby et al., 2010). For instance, in healthy children of comparable age, the multi-center study from the Asthma UK Initiative reported sRaw values of 5.5 hPa.s in the group from center 1 and about 12 hPa.s in the group from center 4 (Kirkby et al., 2010). The importance to develop a method to verify the accuracy of sRaw measurements has been stressed (Robinson et al., 2015) and an easy calibration-check capable of comparing different plethysmographs would thus be highly desirable.

The sRaw physical analog may consist in a known volume of gas placed inside the body box and compressed / expanded through a resistor. There are 2 main metrological issues with such designing: the resistor linearity, and the relationship of the volume of gas compressed / expanded (ΔV) to the corresponding pressure change (ΔP). Gas compression/expansion may be associated with temperature variation that depends on the rate of thermal exchange to the surroundings. Slow maneuvers tend to be isothermal, while fast maneuvers tend to achieve adiabatic conditions. Between the extremes, the ΔV – ΔP relationship is frequency dependent, an obviously unsuitable range for the purpose of reproducible equipment calibration check. It is therefore mandatory that the operational frequency range of the analog is determined beforehand.

The aim of the study was to develop a simple physical analog to check the plethysmograph accuracy and reproducibility in measuring sRaw, and determine optimal operational conditions. This implies designing a resistor with suitable linearity, studying the frequency response of the ΔV – ΔP relationship, and testing the analog plethysmographic measurement accuracy and repeatability.

## Methods And Results

The analog-lung is a resistor mounted on a glass jar. A syringe is connected and may be actuated to produce gas compression – decompression and simulate panting. The pressure – flow relationship of the resistor and the thermodynamics of the analog volume were first characterized separately. Plethysmographic measurements were then performed to determine accuracy with reference to nominal values, as well as short term variability and long term repeatability. Because the protocol for plethysmographic assessment depended on determination of analog characteristics, method and result sections were pooled for each part of the study.

### Characterization of the Analog

#### Measuring Equipment

**FIGURE 1:**
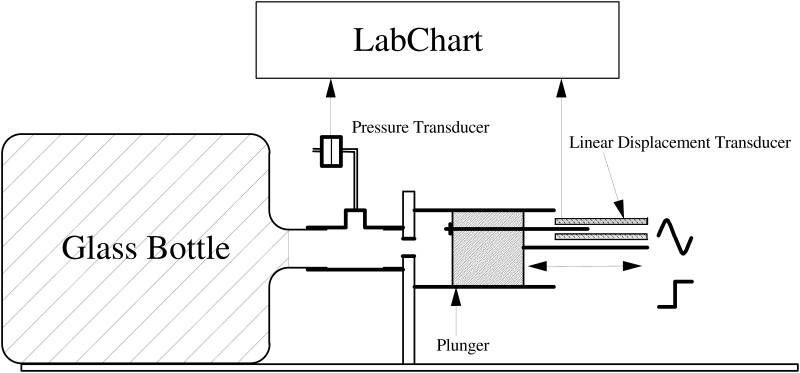
Determination of the thermodynamic characteristics of the analog volume. ΔP is measured by a pressure transducer and ΔV using a linear displacement transducer monitoring the course of the piston pump.

Flow was measured with a Fleisch no 2 pneumotachograph that is linear within ±2.5 l/s (Miller and Pincock, 1986). A reciprocating sinusoidal pump was used to assess the analog ΔV – ΔP relationship. ΔP was measured by a pressure transducer that has a frequency response flat up to 10 Hz (Honeywell 176PC07, Villepinte, France). The course of the syringe plunger was monitored by a displacement transducer (Schaevitz ATA-101, Moorestown, NJ, United States) to measure ΔV (**Figure 1**). Gas compression in the empty jar was studied at frequencies (f) chosen to encompass usual breathing/panting rates (0.25, 0.50, 0.75, 1, 1.5, 2, 2.5, and 3 Hz). Values of ΔV and ΔP were averaged over 5 cycles from each acquisition. The signals were digitized at 200 Hz and recorded by a Labchart recorder (AD instruments Powerlab 16/30, Colorado Springs, CO, United States).

#### Physical Characteristics

##### Resistor

**FIGURE 2:**
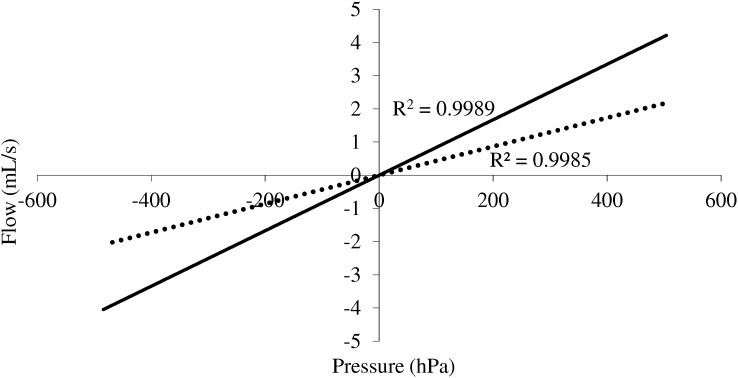
Pressure flow relationship for the 2 resistors tested. The high *r* squared indicates linearity for the practical conditions of the study.

The resistor was made of parallel capillary tubes (0.8 mm inner diameter, 75 mm length) placed in a cylinder. One resistor was made of 122 capillary tubes and the other of 86, in an attempt to simulate a range of airway resistance values representative of lung function testing of young children. The cylinder was mounted in series with the pneumotachograph connected to a source of airflow that was tuned slowly from –0.5 L/s to +0.5 L/s and the pressure drop measured across the resistor. The slope of the pressure – flow relationship computed by regression analysis provided the value of the resistor, and linearity was assessed from the r-squared for that regression. Typical pressure – flow curves are illustrated in **Figure 2**. Linearity of each resistor within 1 L/s peak to peak was attested by the r squared value better than 0.99. The plot of the residuals around the regression equation indicated no significant departure from the regression line for Ra1, while a trend was observed for Ra2 at upper flow values, but the effect was small within the working pressure range (**Supplementary Figure S1**). The resistors had respective nominal values of 4.3 hPa.s/L and 8.2 hPa.s/L.

##### Analog volume

The glass jar has a diameter of 155 mm and length to opening of 250 mm. The reference volume (V) determined by water displacement, and corrected for the volume of connecting tubing, is 2.92 L. Where relevant, the volume of steel wool (39.3 ml) added to achieve isothermal gas compression was subtracted. Airtightness was attested by a mechanical time constant larger than 10 min, i.e., far greater than the thermal time constant (τ_th_) expected for that volume. τ_th_ was then measured from the pressure decay following a quick – 50 ms 10 to 90% rise time – step change in volume of about 10 ml.

The general law governing gas compression/decompression used to compute V from the ΔV – ΔP relation is given by Peslin, (1984):

$$V=-\beta *\left(PB-PH_{2}O\right)*\Delta V/\nabla P\;\;\;\;\;\left(1\right)$$

where PB and PH_2_O are respectively, barometric pressure and partial pressure of water vapor, and β is approximated by:

$$\beta ≃{\left(1+\gamma^{2}\tau_{th}{}^{2}\omega^{2}\right)}^{0.5}/{\left(1+\tau_{th}{}^{2}\omega^{2}\right)}^{0.5}\;\;\;\;\;\left(2\right)$$

Where γ, the ratio of specific heat is 1.40 for air and ω is angular velocity (2^∗^π^∗^f). β is 1 when τ_th_ is zero, i.e., under isothermal conditions where Boyle’s law applies. With increasing frequencies, β increases up to 1.40 when gas compression is adiabatic. In the range of intermediate frequencies – that depend on the analog τ_th_ – the polytropic compression is characterized by frequency dependence of β.

**FIGURE 3:**
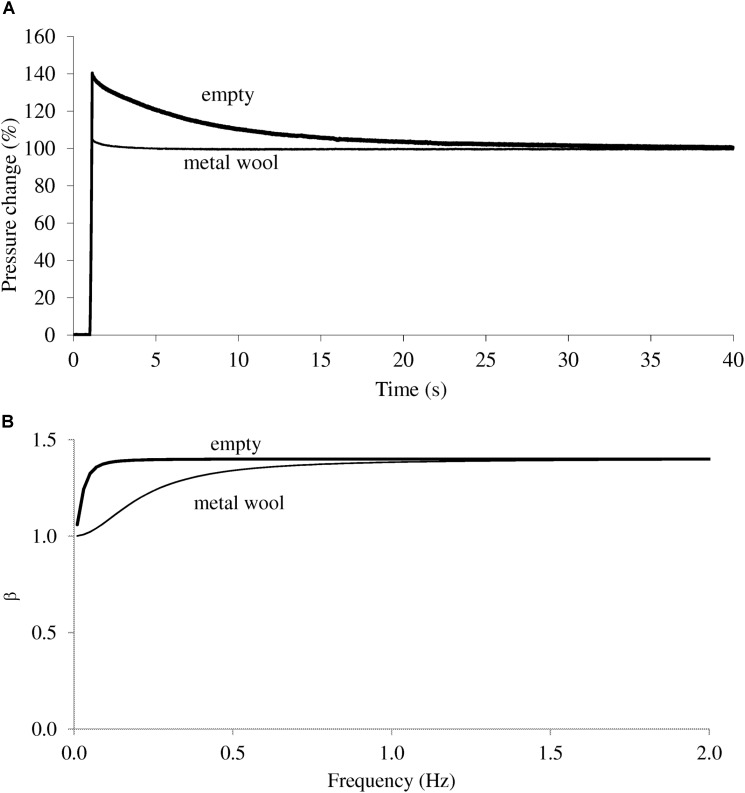
**(A)** Pressure response to a step change in volume. For clarity, ΔP is expressed with reference to the plateau, i.e., the pressure at least 1 min into the acquisition. In the empty jar (heavy trace), the peak pressure is very close to 140%, as expected with adiabatic conditions. The thermal time constant is 6.1 s. When the jar is filled with metal wool, a small spike is still visible soon after the compression maneuver (light trace). The thermal time constant is 0.7 s. **(B)** β, the coefficient governing the general ΔP – ΔV relationship (Eq 2) is computed at frequencies up to 2 Hz in the empty jar (filled symbols) and the jar filled with metal wool (light symbols). Adiabatic conditions – and constant value of β - are obtained at frequencies 0.2 Hz and on, with the empty jar. In contrast, the jar filled with metal wool shows polytropic conditions up to 0.6 Hz.

In the current experimental conditions τ_th_ of the empty jar was 6.1 s (**Figure 3A**), leading to β, computed as in Eq. 2, very close to 1.40 at and above 0.20 Hz (**Figure 3B**). On the other hand, the analog filled with steel wool was found with τ_th_ = 0.7 s (**Figure 3A**) and the computed β was 1 at 0.01 Hz but increased with frequency, especially in the range 0.02 – 0.6 Hz, and β was 1.40 above 1 Hz (**Figure 3B**). Therefore, the effect of polytropic gas compression was observed over a larger frequency interval with the jar stuffed with steel wool than when empty (**Figure 3B**).

**FIGURE 4:**
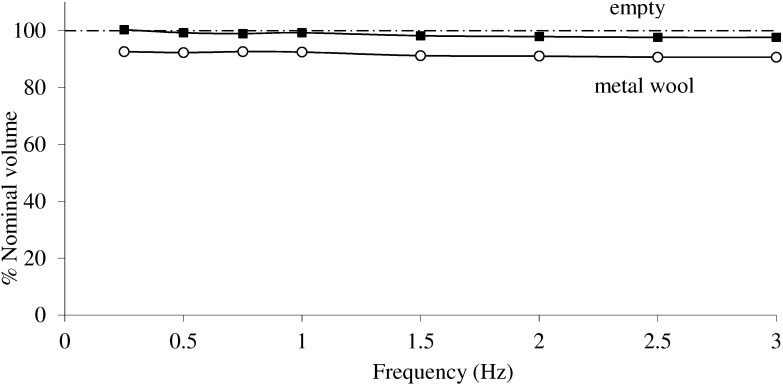
The analog volume is measured at different frequencies using the apparatus described in **Figure 1**, and expressed as % of the nominal value. Less frequency dependence and more accurate estimation are observed when the jar is empty (filled squares) than when filled with metal wool (open squares).

The frequency response of V measured with the reciprocating pump described in **Figure 1** is presented in **Figure 4**. A small frequency dependence of V while empty was observed in the lower frequency domain, but the estimate was within 3% of the nominal value over the whole frequency interval. In contrast, V when the analog was stuffed with steel wool showed somewhat more frequency dependence and a value *ca* 7% below the nominal value (**Figure 4**).

Altogether, there appears to be more underestimation of the analog volume and frequency dependence in conditions where isothermal compression is attempted, i.e., the jar is stuffed with steel wool, than when it is empty. Therefore further testing in the plethysmograph to simulate calibration check prior to clinical measurements was done with the empty jar, at frequencies where gas compression is expected to be adiabatic, and V computed from Eq. 1 using β = 1.40.

### Plethysmographic Measurements

Measurements were obtained in a Jaeger MedGraphics 1085 body box. The barometric plethysmograph has an 8–10 s mechanical time constant and had been reequipped with transducers, electronics, filtering, acquisition procedures, and mathematical handling as previously described and validated (Peslin et al., 1987). Of note, the algorithm to analyze the plethysmograph volume – flow relationship when sRaw is determined has no thermal artifact correction. The daily quality control procedure includes adjusting the gain of the pressure transducer against a water manometer, calibrating the pneumotachograph by the integral method and the plethysmograph volume signal using the built-in 50 mL reciprocating pump.

During the measurement, a technician sits in the plethysmograph, wearing a nose clip. The analog is coupled to the resistor and the breathing apparatus through a T connector. A syringe mounted on the vertical branch of the T allows compression – expansion of the analog volume. Measurements are taken in conditions similar to the routine protocol. Once thermal equilibrium has been reached, the manipulator remains apneic while the syringe is stroked continually, first with the shutter open for 2 s to determine the analog specific resistance (sRa1 and sRa2), and then closed for another 2 s to estimate the analog volume (Va). As indicated above, care was taken to obtain measurements at frequencies around 2 Hz where compression is adiabatic, and the flow range during the mock panting was limited to within 1 L/sec peak to peak.

The resulting Va, sRa1, and sRa2 allowed calculation of corresponding analog resistances (Ra1 and Ra2). Data are expressed as percentage of the relevant reference (see above). The reference volume was corrected for the 68 ml tubing connection upstream to the shutter. PB ranged 726–730 mmHg and PH_2_O about 10 mmHg during the period of the experiments.

Measurements were carried out on at least 4 different days, each daily set consisting in 5 maneuvers. Five sets were obtained with resistor 4.3 hPa.s/L and 4 with resistor 8.2 hPa.s/L.

The main results are illustrated in **Table 1** that shows reasonable accuracy for Va and sRa determinations that are, on average, within 5% of the nominal value with both analogs. Accuracy is equally good for Ra2, but Ra1 appears slightly underestimated. The between – session coefficient of repeatability is within 5% for all estimates of Va, sRa, and Ra. The within session coefficient of variation is in a similar range, except for sRa1 and Ra1 which is around 6%.

**Table 1:** Characteristics of 2 physical analogs retrieved from daily plethysmographic measurements.

	Volume		Resistor					
	*Va*				*sRa*		*Ra*	
	% ^∗^	Cov %	freq (Hz)	V’(L/sec)	% ^∗^	cov %	% ^∗^	Cov %
**Analog1°**								
1	107	2.2	2.80	0.69	94	7.7	89	8.6
2	107	2.9	2.84	0.77	103	10.5	97	12.0
3	103	1.8	3.00	0.86	98	3.8	95	3.3
4	104	2.7	2.90	0.87	95	3.2	93	1.7
5	103	2.0	3.10	0.86	94	3.3	92	4.7
*Mean (SD)*	*105 (2.0)*	*2.3*	*2.9 (0.1)*	*0.8 (0.1)*	*97 (3.8)*	*5.7*	*93 (3.4)*	*6.1*
*RC*	*1.9*				*3.9*		*3.6*	
**Analog 2^∘^**					
1	107	1.6	3.15	0.75	105	3.4	100	2.9
2	103	1.4	3.06	0.85	103	3.7	101	2.8
3	103	2.2	3.08	0.75	100	1.2	99	2.5
4	105	1.1	3.20	0.99	106	2.8	102	3.3
*Mean (SD)*	*104 (1.9)*	*1.6*	*3.1 (0.1)*	*0.8 (0.1)*	*104 (2.4)*	*2.8*	*100 (1.2)*	*2.9*
*RC*	*1.8*				*2.3*		*1.2*	

*°Analog 1 and 2 are made of respectively, 122 and 86 capillaries. See methods. Va, estimated analog volume; sRa, Ra, respectively, estimated analog specific resistance and analog resistance; freq, V’, respectively, frequency and flow range during sRa measurement; ^∗^ %, of nominal value; Cov, within session coefficient of variation; RC, between session repeatability coefficient*.

## Discussion

The design of a physical analog to test plethsymographic equipments to measure sRaw and thoracic gas volume appears rather straightforward when considering the physical laws involved. An important condition when designing the resistor is linearity within the operational range of flow, so as to insure reproducible measurements. We found that the use of a length of parallel capillary tubes allowed designing resistors whose nominal values were representative of those encountered in pediatric lung function testing. A close look at the pressure-flow regression line of sRa2 could detect some deviation from linearity around 0.4 L/sec (i.e., 0.8 L/sec peak to peak). However, the effect was small within the working range and the device still compatible with the practical objective of this study.

The gas compression – decompression occurring within the real lung is assumed to be isothermal. Within an individual alveolus, it is estimated that the ratio of alveolar gas volume to alveolar wall surface area is highly favorable to complete heat exchange and isothermal conditions resulting in negligible τ_th_ (Peslin, 1984). Ideally therefore, a lung analog for plethysmographic measurements should reproduce such conditions where the pressure – volume relationship obeys Boyle’s law (i.e., β is 1 in Eq. 1). τ_th_ depends essentially on the volume of gas inside the container, and metal wool that has a high thermal conductivity is added in an attempt to reproduce isothermal conditions (Reinmann et al., 2001). We could verify however, that a thermal time constant could still be identified, and isothermal gas compression could be assumed only when frequency was as low as 0.01 Hz. A probable cause may be the unequal repartition of metal wool leading to inhomogeneous distribution of thermal time constants. Negative frequency dependence of the analog stuffed with metal wool was found up to about 1 Hz and there was some underestimation of V. The observed frequency dependence was likely to reflect the polytropic gas compression, as expected from Eq. 2. A previous work on assessment of an infant mechanical lung model similarly reported the difficulty to achieve proper isothermal gas compression, together with negative frequency dependence and some underestimation of the analog volume (Frey et al., 2001). On the other hand, with the empty jar, adiabatic compression could be predicted at frequency 0.20 Hz and above. Frequency dependence was negligible and the error in predicting the analog was quite small (**Figure 4**). Altogether, we found in these experiments as well as in previous studies that isothermal condition was quite difficult to insure. In other words the isothermal conditions that readily occur within the lung due to a very large number of very small airspaces cannot be reproduced in this physical model. It is not feasible to insure a null τ_th_ except at very low frequencies, non-practical for the purpose of routine equipment testing.

When comparing different plethysmographic equipments devoted to pediatric lung function, a simple analog whose mechanical characteristics are within the range of those measured for sRaw in children is important, but isothermal gas compression as occurring within the alveoli is not mandatory. The important criterion is to obtain conditions that would insure reproducible inter-laboratory measurements. The study clearly establishes that at or above 2 Hz, the adiabatic compression may be guaranteed with the current analog volume. Using such range of frequency, the overall accuracy of plethysmographic volume measurements mimicking conditions of lung function testing was found within 105% of the nominal value. Within session coefficients of variability as well as between session coefficients of repeatability were in the order of 2%, i.e., rather small when compared to the range reported under clinical conditions. For instance measurements of thoracic gas volume in non-naïve adult subjects resulted in within session variability of 5% and day to day repeatability of about 6% (Peslin et al., 1987). The sRa1 and sRa2 were respectively, 97 and 104% of corresponding nominal values, thus showing similar measurement accuracy than observed for Va (**Table 1**). The within session variability and between session repeatability were within 3% for sRa2 but somewhat larger for sRa1 especially the within session variability. We believe this is likely due to less favorable signal to noise ratio when the sRa1 is measured because a smaller ΔV is generated during the maneuvers compared to sRa2. Nevertheless these figures are within those reported for sRaw in non-naïve adult subjects, i.e., 10% variability and 20% between day repeatability (Peslin et al., 1987). Also, the estimated short term coefficient of variation for either sRa here is well within those reported for clinical measurements of sRaw – about 10% – in healthy or asthmatic children (Bisgaard and Klug, 1995; Klug and Bisgaard, 1997; Coutier et al., 2015; Ioan et al., 2016). The between session repeatability was within 5% for all analog parameters, an important result in the context of between laboratory equipment comparison, while the between day variability in healthy adult subjects was reported to be respectively, about 6% for thoracic gas volume and 20% for sRaw (Peslin et al., 1987).

It is concluded that a physical analog to check the calibration of a plethysmograph may easily be designed from simple material, not requiring metal wool provided the mock thoracic gas volume – sRaw maneuver is performed at frequency ≥2 Hz and adiabatic conditions are accounted for (Eqs. 1–2). It is surmised that the procedure should improve comparability among different laboratories and should thus be helpful to international multi-center studies of pediatric respiratory diseases.

## Author Contributions

BD, II, CD, CS, FM, and SD-A have prepared the project of this study. BD, CB, and CD performed lung function test, BD, CB, and CD performed data collection and statistics. BD, II, CD, CS, FM, and SD-A prepared the draft of the manuscript. BD, II, CD, CS, FM, and SD-A completed the work and revised the final manuscript.

## Conflict of Interest Statement

The authors declare that the research was conducted in the absence of any commercial or financial relationships that could be construed as a potential conflict of interest.

## Supplementary Material

FIGURE S1 Residuals around the regression equation of pressure to flow are plotted against flow. The small resistor (Ra1) shows values close to zero. The deviation observed at large flow with Ra2 is small within the working pressure range.
