# Supplementary figures and images for: A Calibration Device to Compare Body Plethysmographs Among Pediatric Lung Function Laboratories
Source: Front Physiol. 2018 Oct 9;9:1408. doi: 10.3389/fphys.2018.01408 (PMC6189406; doi:10.3389/fphys.2018.01408)

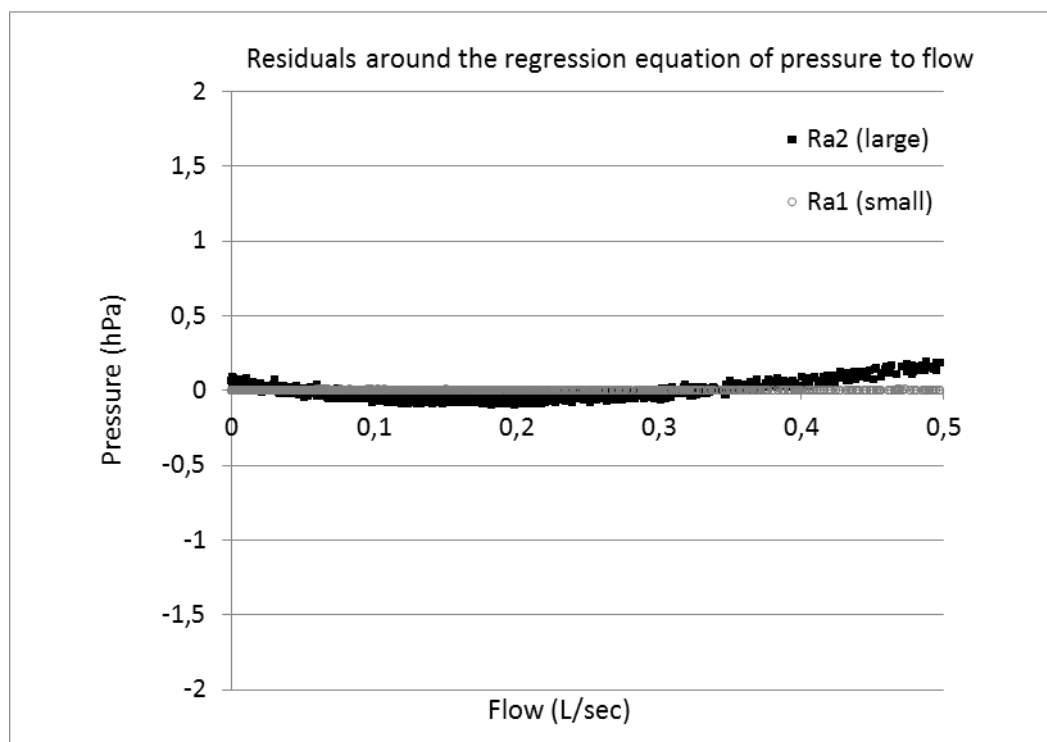

Supplement: FIGURE S1 — Residuals around the regression equation of pressure to flow are plotted against flow. The small resistor (Ra1) shows values close to zero. The deviation observed at large flow with Ra2 is small within the working pressure range. [file Data_Sheet_1.PDF]
